# Reconciling Contemporary Approaches to School Attendance and School Absenteeism: Toward Promotion and Nimble Response, Global Policy Review and Implementation, and Future Adaptability (Part 2)

**DOI:** 10.3389/fpsyg.2019.02605

**Published:** 2019-11-29

**Authors:** Christopher A. Kearney, Carolina Gonzálvez, Patricia A. Graczyk, Mirae J. Fornander

**Affiliations:** ^1^Department of Psychology, University of Nevada, Las Vegas, Las Vegas, NV, United States; ^2^Department of Developmental Psychology and Teaching, Universidad de Alicante, San Vicente del Raspeig, Spain; ^3^Department of Psychiatry, University of Illinois at Chicago, Chicago, IL, United States

**Keywords:** school attendance, school absenteeism, truancy, school refusal, school withdrawal, school exclusion, multi-tiered system of supports, response to intervention

## Abstract

As noted in Part 1 of this two-part review, school attendance is an important foundational competency for children and adolescents, and school absenteeism has been linked to myriad short- and long-term negative consequences, even into adulthood. Categorical and dimensional approaches for this population have been developed. This article (Part 2 of a two-part review) discusses compatibilities of categorical and dimensional approaches for school attendance and school absenteeism and how these approaches can inform one another. The article also poses a multidimensional multi-tiered system of supports pyramid model as a mechanism for reconciling these approaches, promoting school attendance (and/or prevention of school absenteeism), establishing early warning systems for nimble response to school attendance problems, assisting with global policy review and dissemination and implementation, and adapting to future changes in education and technology.

## Introduction

The field of school attendance and absenteeism (SA/A) remains, as it has always been, at various crossroads. Categorical and dimensional approaches to conceptualizing SA/A are manifold, and each approach has its own validity for defining, classifying, and providing assessment and prevention/intervention recommendations for this population (see Part 1 of this two-part review; Kearney et al., 2019). Categories generally refer to dichotomies and distinctions to identify groups, whereas dimensions generally refer to fluid or latent constructs arranged along various spectra or continua. Key categorical dichotomies and distinctions of SA/A include school refusal-truancy, excused-unexcused absences, school withdrawal and school exclusion, acute-chronic duration, and diagnostic categories. Key dimensional aspects of SA/A include defining school attendance and its problems along a continuum, multi-tiered system of supports for preventative and intervention strategies arranged according to student need, risk/contextual factors, absenteeism severity, developmental level, and functional profiles of school attendance problems.

The development of categorical and dimensional approaches to better understand a particular phenomenon is not unique to the field of SA/A; indeed, such bifurcation is a common aspect of the study of many different child behavior problems such as anxiety and mood disorders, developmental disorders, and attention-deficit/hyperactivity and conduct disorders (Hankin et al., 2011; Ghio et al., 2015; Wakschlag et al., 2015; Elton et al., 2016; Sprafkin et al., 2016). A key task moving forward will be to draw from the validity of all approaches to design a framework for SA/A that can facilitate the promotion of school attendance, nimble responses to emerging school absenteeism, effective policy review across jurisdictions, wide dissemination to various locations and settings, and adaptation to future, rapid changes in education and technology.

As noted in Part 1 of this review, Coghill and Sonuga-Barke (2012) stated that both categorical and dimensional approaches can coexist within a given phenomenon by serving different but equally useful purposes. Both categorical and dimensional approaches can be applied to a given heterogeneous construct. Categories are useful for providing general rules and cut-points for distinguishing broad groups of behavior, and dimensions are useful for providing data to adjust these cut-points along various spectra such as age, gender, temperament/behavior, developmental level, and setting to improve the categorical rules. Categorical distinctions can be useful descriptors of a particular current state, and dimensional profiles can be used to determine if that categorical state changes in degree of intensity (e.g., to nonproblematic or to more problematic) over time to inform treatment, longitudinal, and prognostic analyses. Categories and dimensions together can thus form a synergistic and breathable system that allows for considerable adaptation to future scientific and other advances (Hudziak et al., 2007).

Over the next sections of this article (Part 2 of a two-part review), we discuss a possible pathway toward reconciling contemporary categorical and dimensional approaches to SA/A. This discussion initially involves sample compatibilities across extant categories and dimensions of SA/A and how these constructs might be blended or matched with one another. This section focuses on pertinent or prominent examples and is not an exhaustive review of all possible affinities. This discussion then includes a multidimensional, multi-tiered system of supports (MTSS) pyramid model that may be used as a framework to include various categorical-dimensional aspects of SA/A. Finally, as mentioned, we explore how such a model could enhance promotion of school attendance and/or prevention of school absenteeism, expedite nimble clinical and other responses to emerging absenteeism *via* early warning system development, assist in policy review and dissemination across jurisdictions and disciplines, and adapt to future and rapid changes in education and technology. We emphasize that the framework presented here is a heuristic one, not meant to be necessarily optimal or capstone in nature, but rather one designed to help spur the field toward reconciliation, common language, and advancement. We fully expect and hope that the framework will evolve over time.

## Compatibilities of Categories and Dimensions of School Attendance and Absenteeism

Compatibilities of categories and dimensions of SA/A (described in Part 1 of this two-part review) can be described in two main ways. First, many categorical approaches for SA/A actually have many dimensional features, and many dimensional approaches for SA/A actually have many categorical features. Second, many categorical and dimensional approaches for SA/A have striking similarities that may indicate general agreement about a particular construct and refer to that construct from somewhat different perspectives. The examples provided next include both ways of describing compatibilities among categories and dimensions of SA/A.

### Categories of School Attendance and Absenteeism With Dimensional Features

As mentioned in Part 1 of this review (p. 3), truancy is one of the most venerable constructs in the field of SA/A. From a categorical perspective, truancy may refer to illegal, unexcused school absence without parental knowledge or sanction (Gentle-Genitty et al., 2015). From a dimensional perspective, as noted in Part 1 of this review (p. 4), researchers have found many profiles of truancy along academic status, disability, location, race/ethnicity, in- and out-of-school activities, individual-group-orientation, premediated-spontaneous initiation, and parental academic involvement, among many other variables. Gentle-Genitty et al. (2015) noted as well that categorical definitions of truancy often involve dimensions of absenteeism along time such as arriving late to school, missing a class, and missing a full school day, similar to the definitional spectrum of SA/A presented in Part 1 (p. 7).

Truancy as a category and truancy as a multidimensional construct are compatible notions. A categorical premise of lack of parental knowledge and sanction in truancy, for example, can be informed by various dimensional subtypes to boost its validity and enhance a greater intricacy to this distinction. For example, Keppens and Spruyt (2017) found that parental knowledge of a truant event was a highly nuanced construct that reflected lack of parental knowledge with expectation of parent distress (41.7%), lack of parental knowledge without expectation of parent distress (5.7%), parental knowledge with approval (34.5%), and parental knowledge without approval (18.1%). Truancy as a categorical and dimensional construct is also represented in research regarding forms and functions of SA/A. Researchers who study SA/A categorically generally examine forms of truant behavior such as externalizing problems, whereas researchers who study SA/A dimensionally generally examine functions or factors that maintain school refusal behavior such as pursuit of tangible rewards outside of school (Haight et al., 2011; Iverson et al., 2018; Walter et al., 2018). Both research avenues, however, gravitate toward older youth with less school-based anxiety (Dembo et al., 2016).

As mentioned in Part 1 of this review (p. 3), school refusal often refers to another child-initiated form of school absenteeism. From a categorical perspective, school refusal may refer to emotional distress and reluctance to attend school (Elliott and Place, 2019). From a dimensional perspective, as noted in Part 1 (p. 4), researchers have found many profiles of school refusal along various spectra (e.g., Finning et al., 2018, 2019). Gallé-Tessonneau and Gana (2018), for example, found several main clusters of youth with school refusal involving anxiety and fear of confrontation, adolescent-parent relationships, interpersonal relationship difficulties, and coping difficulties that associated closely with functional dimensions or profiles. Researchers who study SA/A categorically generally examine forms of behavior such as anxiety, depression, and somatic complaints (Jones et al., 2019). Researchers who study SA/A dimensionally generally examine functions or factors that maintain school refusal behavior such as avoidance of negative affectivity and escape from aversive social and/or evaluative situations (Haight et al., 2011; Richards and Hadwin, 2011). Both research avenues, however, gravitate toward youth with more school-based distress (Havik et al., 2015).

Other categorical constructs for SA/A also have dimensional features. For example, the construct of school withdrawal, or parent-initiated school absenteeism, includes a spectrum of parent behaviors such as knowledge, acquiescence, consent, approval, and accommodation, or more passive to more active responses (Kearney and Albano, 2018; Marin et al., 2019). Similarly, school exclusion or school-initiated absenteeism can involve a spectrum of lawful or unlawful administrative responses such as loss of privileges, early school departure, detention, in-school suspension, out-of-school suspension, restorative or other interventions in another location, alternative educational placement, and expulsion as well as duration of the exclusion (Valdebenito et al., 2018). In addition, Birioukov (2016) sought to reframe the categorical dichotomy of excused-unexcused absences along broader distinctions (i.e., voluntary and involuntary) with varying explanations. Voluntary absence, for example, might encompass more student agency involving spectra along motivation to attend school and perceptions of school as a hostile environment. Involuntary absence might encompass more contextual influences that affect a student’s ability to attend school and include spectra along life conditions, opportunities for academic advancement, and access to education (see also Part 1 of this two-part review, p. 5).

### Dimensions of School Attendance and Absenteeism With Categorical Features

As mentioned in Part 1 of this review (p. 10), a functional model of school refusal behavior focuses on dimensions or profiles of the relative strength of maintaining factors for school refusal behavior. The model was originally designed as a clinical strategy to help mental health professionals utilize descriptive and experimental functional analyses to identify a particular prescriptive treatment tailored to these maintaining factors (Kearney and Silverman, 1990). Youth may refuse to attend school to (1) avoid school-based stimuli that provoke a sense of negative affectivity (anxiety and depression), (2) escape from aversive social and/or evaluative situations at school, (3) pursue attention from significant others, and/or (4) pursue tangible rewards outside the school. The functions were based on wide parameters of negative and positive reinforcement (Kearney, 2001).

In this functional model, a dimensional profile of maintaining factors is derived *via* a comprehensive assessment that includes descriptive measures, rating systems, behavioral observations, and formal hypothesis testing, among other means. Some erroneously equate one descriptive instrument with the broader functional model, but the functional distinctions can be measured in many ways to derive detailed and nuanced clinical profiles of each (Kearney and Tillotson, 1998). Indeed, the functional model was specifically designed to be flexibly applied to different clinical and educational settings to account for differences in local practices as well as the heterogeneity of school attendance problems and to enhance the treatment utility of assessment (Nelson-Gray, 2003). With respect to the latter, a primary function based on relative strength to the others may be categorically chosen as a starting point for prescriptive intervention (Kearney and Silverman, 1999). A categorical nature of the functional model is further reflected in research work examining differences between the functions (e.g., Haight et al., 2011). As such, the model is a flexible, prototypical categorical-dimensional approach for SA/A and has been generally utilized and studied in this manner (e.g., Lyon and Cotler, 2009; Gresham et al., 2013; Nuttall and Woods, 2013; Elsherbiny, 2017).

Similarly, a multi-tiered system of supports (MTSS) model of SA/A (see Part 1 of this review, pp. 7–9) involves several dimensional continua with respect to absenteeism chronicity and severity as well as degree of risk and contextual factors generally associated with increasingly higher levels of absenteeism. An MTSS model of SA/A also assumes a spectrum of needed supports for youth and their families ranging from (1) system-wide or universal preventative approaches to (2) targeted interventions for mild to moderate school attendance problems to (3) intensive interventions for chronic and severe absenteeism (Kearney, 2016). The spectrum-based nature of MTSS is designed in part to enhance feasibility for, and thus applicability to, various educational and other settings (Stoiber and Gettinger, 2016).

A key component of MTSS models, however, is a categorical tier-based structure with ostensibly clear demarcations between each level of supports. Specific demarcations are important for understanding when to shift the focus of intervention to a higher (or lower) tier. Within a reading context, for example, standardized assessment protocols may be utilized to identify students with specific comprehension or word decoding problems that warrant Tier 2 or Tier 3 intervention (Leonard et al., 2019). In addition, teacher-based screening and office disciplinary referrals for behavior may indicate a failed intervention and thus a marker for movement to a different tier (Naser et al., 2018). As such, assessment profiles inform movement from one categorical tier to another. With respect to an MTSS model for SA/A, identifying when a child could move from one tier to another will involve expanded research into tier-based demarcations that may help inform intervention assignment (Fornander and Kearney, 2019a,b, submitted) (see also later sections).

Other dimensions of SA/A, including those within an MTSS model, have been examined categorically as well. Risk and contextual factors of SA/A, for example, are commonly studied or grouped into child-, parent-, family-, peer-, school-, community-, cultural-, and even government-based distinctions, as well as how these distinctions change across locations (Kearney, 2008; Lamb et al., 2010; Correia and Marques-Pinto, 2016; Şahin et al., 2016). Researchers examine these risk factors *via* spectra of accumulated risk as well as *via* statistical modeling to compare the contributed risk of each group (Chen et al., 2016; Goodrich et al., 2017; Chung and Lee, 2019; Sansone, 2019). Similarly, researchers have examined absenteeism severity both as dimensional ranges and as categorical distinctions (Skedgell and Kearney, 2016, 2018; Stempel et al., 2017).

### Categories and Dimensions of School Attendance and Absenteeism: Informing One Another

Categorical and dimensional approaches to SA/A have many compatibilities as well as overlapping qualities and purposes. As noted earlier, categorical distinctions of SA/A, which have traditionally suffered from considerable ambiguity and limited construct validity (Part 1 of this review, p. 6), may be better informed by common and empirically based higher-order dimensions. Such dimensions may help identify functional analytic and temporal aspects to improve the practical nature of different categories in clinical and educational practice (Brown and Barlow, 2009). For example, identifying risk or behavioral marker profiles would help improve a distinction between Tier 1 prevention and Tier 2 early intervention (Mitchell et al., 2011). In addition, identifying specific pathognomonic or at least assident features of various SA/A categories may ultimately come from examining ranges or profiles of constructs such as avoidance, emotion regulation, cognitive features, temperament, parent responses, family environment dynamics, association with deviant peers, school climate, and perhaps even biopsychosocial or bioecological aspects (Caron et al., 2006; Rothbart and Posner, 2015; Gottfried and Gee, 2017). In the next section, we posit a multidimensional multi-tiered system of supports pyramid model of SA/A that allows space to explore these research avenues while simultaneously charting preventative and intervention processes for immediate dissemination and implementation.

## A Multidimensional Multi-Tiered System of Supports Pyramid

Multi-tiered system of support (MTSS) models, including Response to Intervention and Positive Behavioral Interventions and Supports/School-wide Positive Behavior Support, are often represented *via* one-dimensional triangles as illustrated in Part 1 of this review (p. 8). As discussed, these approaches represent multiple tiers of preventative and intervention strategies for various academic, social, and behavioral issues. These tiers are arranged along a continuum of needs of support targeted toward all students (prevention), some percentage of students (early intervention), and some lesser percentage of students (intensive intervention). Kearney and Graczyk (2014) were the first to apply these principles to SA/A (see Part 1 of this review for greater detail, pp. 7–9).

A key constraint of the one-dimensional triangle representation of MTSS is that it assumes considerable homogeneity among the population at hand, such as all children in a particular elementary school who are learning to read or all adolescents in a particular high school with a disruptive behavior resulting in an office disciplinary referral (Sugai and Horner, 2009). As such, preventative and intervention strategies are usually geared in similar fashion, albeit with some flexibility based on nuanced factors such as the function of misbehavior, intensity of punitive response, and responding administrator (e.g., teacher and dean) (Crone et al., 2015). Such an approach appears reasonable at Tier 1 where the focus is on promoting a certain phenomenon (e.g., ability to read) and/or preventing a certain phenomenon (e.g., classroom disruption) for all (and generally similar) students in a given setting. The use of communal approaches at Tier 2 and Tier 3, however, may be less efficacious for as heterogeneous and complex a population as students with school attendance problems.

A progressive conceptual framework for an MTSS approach is to emphasize the notion of a multi-dimensional (and thus multi-sided) pyramid to account for greater heterogeneity as well as clinical and research avenues for a certain population (Dulaney et al., 2013). An example is a multi-tiered, multi-domain system of supports (MTMDSS) model (Hatch et al., 2018). In an MTMDSS model, various tiers of support are associated with multiple domains such as school counselor efforts to address, simultaneously and yet differently, the academic, career readiness, and social/emotional needs of their students (Hatch et al., 2019). These tiers of support remain similar to the three levels of an MTSS model but the presence of multiple sides means the tiers can apply variously and flexibly to different domains.

**Figure 1:**
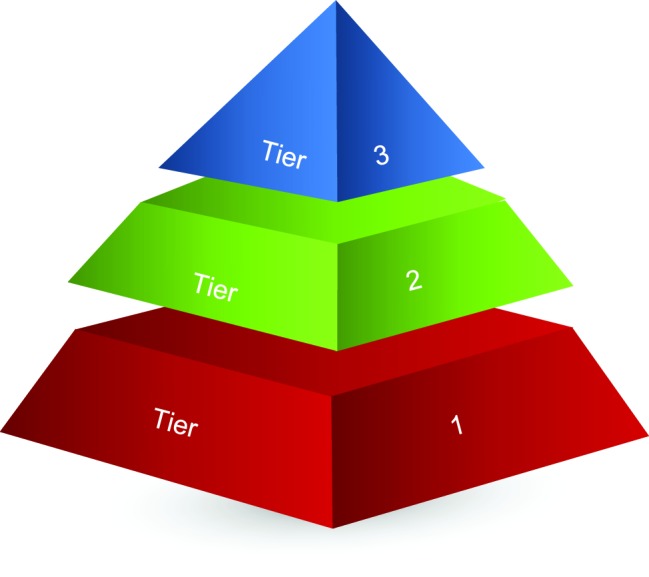
Illustration of a sample multidimensional multi-tiered system of supports pyramid model for school attendance and school absenteeism.

The basic conceptual structure of a multi-dimensional pyramid may fit well with the multifaceted nature of SA/A. In this structure (Figure 1), different sides of a multi-dimensional pyramid could reflect different sets of key categorical-dimensional domains of SA/A. Such domains, among many others, could involve (1) child-, parent-, or school-initiated/oriented school attendance problems, (2) different dimensions of categories such as truancy, (3) functional or risk and protective factor profiles or clusters, (4) school attendance problems in preschool, elementary, middle, and high school students, and (5) schools at low, medium, and high risk for absenteeism. In addition, multi-dimensional pyramids could be developed and tailored to individual jurisdictions with different set points for movement across the tiers. Such pyramids would also allow for better cross-disciplinary work and enhance creativity and innovation about how this population is conceptualized. A multi-dimensional pyramid could vary according to the number of domains desired (e.g., four and six sides) as well. Most importantly, this approach mandates the development of preventative and intervention strategies for each tier no matter what domains are used.

As an example, Lyon and Cotler (2009) juxtaposed functional dimensions along microsystem, mesosystem, and exosystem levels of intervention for school refusal behavior. Microsystem interventions address more direct, proximal, or immediate influences on school attendance problems, and specific aspects within the microsystem can be linked to specific functional dimensions. In this framework, (1) peer microsystem interventions (e.g., mentoring and social skills) might best be linked to avoidance of social/evaluative situations and pursuit of tangible reinforcement; (2) family microsystem interventions (e.g., contingency management and contracting) might best be linked to avoidance of social/evaluative situations, pursuit of parental attention, and pursuit of tangible reinforcement; and (3) school microsystem interventions (e.g., incentive programs and academic support) might best be linked to avoidance of negative affectivity, avoidance of social/evaluative situations, and pursuit of tangible reinforcement.

Mesosystem interventions address connections between settings most relevant to a child such as parent-school official contacts. In this framework, mesosystem interventions (e.g., school engagement and parental involvement initiatives) might best be linked to pursuit of parental attention and pursuit of tangible reinforcement. Exosystem interventions (e.g., policy changes and statutes) address more distal social structures or settings that have an indirect influence on school attendance problems and may best be linked to all functions of school refusal behavior. The authors also discussed macrosystem influences, or societal or cultural/subcultural influences that envelop other levels (in this case, those involving school absenteeism). Such influences may include, for example, shifts in economic opportunities, globalization, migration/immigration, and labor markets that impact school dropout rates (Brewer and McEwan, 2010; Coxhead and Shrestha, 2017).

Lyon and Cotler’s (2009) approach, a key prelude to the multi-tiered frameworks discussed here and in other articles (see also Lyon and Bruns, 2019), emphasized the notion of multifaceted tiers that each reflected multiple domains related to school attendance such as functional profiles, contextual factors, and intervention types and levels. In addition, the authors worked to supersede traditional notions of school refusal and truancy, emphasize how multi-systemic interventions can augment personalized clinical treatment approaches, and encourage the expansion of tailored strategies to best serve different ethnic and cultural groups, a process that remains largely underdeveloped in the SA/A field even today. One omission of Lyon and Cotler’s (2009) approach was the notion of preventative practices to proactively address multi-system factors leading to school attendance problems, a topic we turn to next.

### Base of the Pyramid: Promoting School Attendance

The notion of a multidimensional MTSS/MTMDSS pyramid model carries some potential advantages as a heuristic for SA/A. First, the notion of a multidimensional pyramid implies a common base involving children and adolescents who are attending school without difficulty. The base of a pyramid is necessarily broad and strong and critical for the support of the upper tiers. As such, the base of the pyramid is the most fundamental aspect of the structure and must be well maintained. The notion of a pyramidal base thus means that all stakeholders in the field of SA/A begin with the common premise that school attendance is valued and that promoting school attendance (and/or preventing school absenteeism) must be the foundation for all other efforts in this area.

Second, the notion of a strong (and larger) pyramidal base means that most efforts in this area will need to focus on promoting school attendance and not simply on reducing absenteeism. With respect to SA/A, this means that school districts, health and mental health professionals, and lay persons must invest significant resources and efforts into Tier 1 practices to prevent youth from entering Tiers 2 and 3. All too often, stakeholders in this field concentrate on policies, procedures, sanctions, treatments, and other methods to react to student absenteeism as opposed to engaging in measures to proactively maintain and boost school attendance. The notion of a multidimensional base means that proactive, preventative efforts must be emphasized and can be tailored to individual schools, jurisdictions, and cultures.

Third, the notion of a strong pyramidal base means that researchers must focus as much on protective and promotional factors toward high school completion (or its equivalent) as on risk factors and other aspects of school absenteeism. Some continue to invest heavily in incremental distinctions of youth with school absenteeism with little investment toward identifying those who do complete school. Indeed, the absence of risk is not the same as the presence of growth. In addition, many researchers tend to focus on the negative consequences of school absenteeism and dropout and less so on the benefits of graduation. A better understanding of such protective factors would greatly inform prevention science in this and related areas (Kieling et al., 2011; Lösel and Farrington, 2012).

Zaff et al. (2017) reviewed literature on factors that promote high school graduation, with a particular focus on dimensions of positive youth development as well as proximal and distal influences within a student’s ecology. Such protective and promotive factors included malleable assets, or those potentially sensitive to intervention, and upstream factors, or those more systemic and likely more difficult to modify. The authors made an astute point that simple lack of risk factors in a particular child does not necessarily imply that the child is thriving or that development is optimized. Instead, researchers and others must focus on variables that actively promote educational attainment, not simply on those that predict school absenteeism and dropout.

Individual student factors found most to predict high school graduation or continued school enrollment included intrinsic motivation to achieve positive educational outcomes, enhanced school engagement, student expectations for academic attainment, and internal locus of control. School engagement can come in many forms, and the authors found that high levels of behavioral (e.g., attending school and completing assignments), emotional (e.g., connection with school and enjoying school), and cognitive (e.g., strategic learning and intellectual curiosity) were most related to academic success and graduation. Of these variables, particularly salient predictors included attendance, social and academic engagement, and arts and athletic participation. Expectations for, and perceived control of, positive academic outcomes were potent predictors as well. Effect sizes were small to moderate.

Parent factors found most to predict high school graduation or continued school enrollment included parental academic involvement and parent-child connection. The former may be associated with attending school-based meetings and conferences, participating in school-based organizations, communicating regularly with school officials, assisting with homework, and setting clear rules about homework and maintaining a good grade point average. Many of these effects remained even after controlling for demographic and school composition variables. Parental social support and regular parent-child communication comprised the parent-child connection construct. Effect sizes for parent influences were generally small. Peer-related factors were more limited and included positive peer norms, or expectations of what behaviors are valued within a particular group of friends. This may include enhanced expectations for maintaining grade point average and for valuing education. Effect sizes for peer influences were generally small.

School-related factors found most to predict high school graduation or continued school enrollment included positive student-teacher relationships, smaller schools, participation in school-based extracurricular activities, and career and technical education. Positive student-teacher relationships can include respectful interactions, teacher interest in students, and student belief in teacher competence. This may relate to smaller schools as well, where teachers and students may be more knowledgeable of one another. Extracurricular activities, including community service participation, may relate specifically to social competence, educational aspirations, and sense of agency among students. Career and technical education opportunities positively impact continued school enrollment in particular. Effects sizes for school variables ranged from small to large.

Finally, the primary community-related factor found most to predict high school graduation or continued school enrollment was participation in out-of-school time programs, or those collections of programs focused on community service, social-emotional learning, and academic enrichment. The authors concluded that more research is needed on how all of these protective factors interact with one another to enhance the trajectory toward graduation, how the factors operate differently across students and contexts, and how risk and demographic factors moderate the effect of assets to promote graduation (Zaff et al., 2017).

Zaff et al.’s (2017) efforts also reveal the value and utility of examining various key dimensions or domains of functioning to inform categorical distinctions between nonproblematic (Tier 1) school attendance and problematic (Tier 2) school absenteeism, and thus preventative targets. Indeed, effective school dropout prevention programs are often based on dimensions of student engagement with school, parental involvement, and school climate (Wilson et al., 2011). In addition, effective components of programs designed to increase school completion are often arranged in dimensional levels of support that involve students (e.g., academic tutoring, social skills instruction, character development, leadership training, work experience, and attendance incentives), schools (e.g., smaller class sizes, anti-bullying, and wider access to mental health support), and policy changes (e.g., reduced stigmatization and use of exclusionary discipline for absenteeism and support for Tier 1 approaches) (Freudenberg and Ruglis, 2007; Freeman and Simonsen, 2015; Balu and Ehrlich, 2018). Utilizing dimensions or domains of functioning to inform categorical distinctions between nonproblematic (Tier 1) school attendance and problematic (Tier 2) school absenteeism also has implications for early warning systems and nimble clinical and other responses to emerging school attendance problems, discussed next.

### Second Tier of the Pyramid: Early Warning and Nimble Response

The notion of a multidimensional MTSS/MTMDSS pyramid model also implies that screening and immediate, nimble response to early warning signs or Tier 2 cases of emerging school absenteeism must be a priority no matter the domain structure utilized on the sides of a pyramid. For example, domains of school attendance problems across elementary, middle, and high school levels must juxtapose with individualized, tailored strategies to identify these problems within the resources and logistical constraints of each domain. This may mean an attendance officer in an elementary school who can call parents immediately each day upon learning of a student absence, a school attendance team (e.g., counselor, dean, and school-based social worker) in a middle school that regularly reviews attendance data and intervenes with a family prior to a legal tripwire for truancy, and an integrated first period teacher-attendance team in high school that coordinates information about attendance, disciplinary referrals, and course grades (Kearney, 2016; Rumberger and Losen, 2017). The ability to nimbly respond to these problems, particularly in school settings, depends heavily on valid early screening methods for SA/A in children and adolescents.

Screening for school attendance problems has occurred in various ways that include both ancillary and direct approaches. With respect to the former, for example, Gall et al. (2000) described a screening process at a school-based health center that included school absence as well as a number of psychosocial and academic variables. Students identified with emotional and behavioral problems and referred for mental health services decreased their school absences nearly 50% and tardiness instances 25%. Mechanisms of action for this effect may include enhanced resilience and health status and behaviors (Walker et al., 2010). Others have screened for ancillary variables such as office disciplinary referrals or health problems such as asthma as markers for attendance problems (Weismuller et al., 2007; Caldarella et al., 2008; Moricca et al., 2013).

Recent endeavors have focused more on direct screening approaches for school attendance problems that include both categorical and dimensional aspects. Early warning systems that focus specifically on attendance, behavioral data/suspensions, and course grades have been found to consistently identify 50–75% of future school dropouts before the event occurred. These categories have been further informed by dimensional data indicating that attendance rates under 85–90%, two or more suspensions, and two or more semester course failures in any subject are particularly pertinent indicators and should be part of a customized multi-tiered response system (Thomas, 2017; Balfanz and Byrnes, 2019). Such data could be collated *via* an online monitoring system, and many school districts utilize software applications to immediately inform parents of an absence as well as course assignments and grades (e.g., https://www.infinitecampus.com/audience/parents-students). Researchers have also utilized text and mobile telephone communications to immediately identify and mitigate school absences (Cook et al., 2017; Smythe-Leistico and Page, 2018) within a dimensional multi-tiered intervention framework.

Other direct screening approaches for school attendance problems focus on spreadsheets listing student demographics, attendance status, behavior, course performance, and interventions (Rumberger and Losen, 2017), brief pediatric consultations (Katz et al., 2016), online self-report methods (Pflug and Schneider, 2016), and checklist methods for categories of absences mixed with level of absenteeism severity (Kearney, 2008; Heyne et al., 2019). A nimble response to a child’s absence from school would benefit from immediate knowledge of whether the absence was due to school exclusion such as suspension or alternative educational placement or home instruction, school-based threat such as bullying, parent-based school withdrawal, legitimate reason such as illness or poor weather, or a child-based anxiety, mood, or conduct problem (Ingul et al., 2019). Basic screening approaches have advantages for limiting the burden on school officials, though early warning systems that are too parsimonious may have limited validity (O’Cummings and Therriault, 2015; Sansone, 2019).

More nuanced early warning systems have thus been developed. Chu et al. (2019) developed an online early detection system for school attendance problems, with a particular focus on teachers, administrative assistants, and school counselors as attendance monitors and trackers. The authors utilized a categorical cutoff of five absences (or 2.78% in a 180-day school year) that included dimensions of absenteeism severity ranging from full days missed to instances of tardiness to early departures from school. School attendance problems were assessed at the end of each of four marking periods throughout the academic year. Yearly absences were more closely associated with an accommodation plan and having a sibling with similar attendance problems. Instances of tardiness were more closely associated with higher grade level, divorced or separated parents, and having a sibling with similar attendance problems. Early departures were more closely associated with male gender, newness to a school, and having a sibling with similar attendance problems.

Several researchers have also recommended machine learning and related predictive modeling methods to study large SA/A-based data sets to help inform such algorithms and early warning systems (do Nascimento et al., 2018). Chung and Lee (2019), for example, utilized random forests in machine learning to predict student dropout among 165,715 Korean students. Key indicators included unauthorized absence, early leave, class absence, and lateness as well as various test scores and school experiences. School dropout was predicted mostly by several risk factors that included all forms of unauthorized school attendance problems. In addition, several protective factors were identified that included self-regulated activity, career development, club activity, and volunteer work. The authors recommended that homeroom teachers utilize such markers to mitigate risk and enhance protective factors *via* appropriate supports and interventions. Indeed, some have advocated for restructuring the role of the homeroom or first-period teacher to quickly identify an absent and transmit the information to a school attendance team member who immediately contacts parents (Lever et al., 2004).

Sansone (2019) also advocated for machine learning approaches to provide algorithms for predicting school dropout among 21,440 ninth-grade students. Key predictors selected by the statistical methods used included age, lack of important math and science courses, grade point average, and whether a student had ever been suspended or expelled from school. Other more secondary predictors included lack of plan to later enroll in college, parent contacted by school about poor attendance, and parent belief that the child will at best attain high school only. The author recommended identifying at-risk students based on these variables to identify effective academic and vocational approaches as well as informing parents of a particular student’s risk level. The author concluded as well that early warning systems that are too parsimonious may lack reliability, and that identifying students at less risk for dropout may be as useful as identifying those at high risk.

More specific to school absenteeism, Kearney and colleagues (Kearney, 2018; Skedgell and Kearney, 2018; Fornander and Kearney, 2019a,b, submitted) conducted several studies utilizing ensemble and classification and regression tree (CART) analyses to identify demographic, academic, behavioral, and family factors that best differentiated school absenteeism at various severity levels. Skedgell and Kearney (2018) examined records from 316,004 students across elementary, middle, and high schools to identify academic and demographic variables that best predicted distinctions between <1 and 1+% absenteeism, <10 and 10+% absenteeism, and <15 and 15+% absenteeism based on differentiations sometimes recommended in the literature.

Four predictors that best differentiated youth at <1 and 1+% absenteeism severity levels included ethnicity (Hispanic, African American, Caucasian, biracial, American Indian, or Pacific Islander), grade point average (0.00–2.00), grade level (1, 2, 9, 10, 11, or 12), and Individualized Education Plan (IEP) eligibility. Three predictors that best differentiated youth at <10 and 10+% absenteeism severity levels included age (>15.5 years), ethnicity, and low grade point average. Four predictors that best differentiated youth at <15 and 15+% absenteeism severity levels included age (>16.5 years), ethnicity, low grade point average, and grade level (1, 6, 7, 8, 10, 11, or 12). *Post hoc* analyses were also conducted for developmental school levels. At the elementary school level, ethnicity and grades 1 and 2 were most predictive of all absenteeism severities. At the middle school level, ethnicity and IEP eligibility were most predictive of <1 and 1+% absenteeism, whereas ethnicity was most predictive of the other absenteeism severity levels. At the high school level, low GPA was most predictive of all absenteeism severity levels.

Fornander and Kearney (2019a,b, submitted) further used ensemble and CART analyses to examine predictors of various absenteeism severity levels (1+, 3+, 5+, 10+, 15+, 20+, 30+, 40+%) in youth with school attendance problems referred for clinical services or to a truancy or family court. As with the demographic and academic variables described in the previous study, predictive risk factors tended to be more homogeneous at higher levels of absenteeism severity. These studies included analyses of family environment variables as well as internalizing symptoms of anxiety and depression.

With respect to family environment, higher levels of absenteeism (i.e., 15+%) were more closely related to lower achievement orientation, active-recreational orientation, cohesion, and expressiveness. Many findings were quite nuanced, however. For example, lower expressiveness was evident at less severe (3, 5%) and more severe (20, 30%) levels of absenteeism, though elevated expressiveness was predictive of 10+% absenteeism. In addition, family cohesion was not predictive at 1+ and 3+% absenteeism but less cohesion was more predictive of higher levels of absenteeism. Elevated conflict was more predictive of 5+% absenteeism severity; whereas lower conflict was more predictive of 10+% absenteeism severity. In addition, less family control was more predictive of higher levels of absenteeism severity (20+, 30+%).

With respect to internalizing symptoms, one consistent item that distinguished levels of higher from lower absenteeism severity was a depression item related to lack of enjoyment. Predictive items at 1 and 3% absenteeism were less informative than items at higher absenteeism levels. For example, endorsement of less anxiety was more predictive of higher levels of absenteeism severity, a finding similar to Skedgell and Kearney (2016) who found that very high levels of absenteeism were generally marked by less anxiety. This could mean that extensive absence from school mitigates anxiety at the time of assessment.

The nascent development of valid early warning systems of SA/A (as well as continuous screening devices) has tremendous potential for informing more nimble responses on the part of school officials. This is especially critical now that schools are a primary site of mental health care for most youth (Green et al., 2013; Lyon et al., 2019). Screening devices with set algorithms or rules would allow for nearly simultaneous assessment and intervention, such as quicker use of informed clinical, referral, and other strategies to mitigate emerging school attendance problems. Such devices may also help school officials triage or narrow the focus of these nimble responses, such as toward child, parent, and peer microsystems (Lyon and Cotler, 2009; Kearney, 2019). The studies also reveal a fine line between parsimony and validity, however, meaning that researchers must thread the needle of identifying informative early warning systems that are acceptable and not burdensome to school-based professionals.

Clusters of variables are likely more useful for deriving an algorithm to inform an early warning system for school attendance problems, including for categories of absences, than singular factors such as child internalizing behavior. Indeed, researchers in child psychopathology increasingly use item response theory and signal detection approaches to identify multiple dimensional spectra of normal and abnormal functioning (White et al., 2017; Wakschlag et al., 2019). These approaches would be particularly useful for identifying cutoffs and criteria, transdiagnostic constructs, and multi-system responses (Nigg, 2017) for school attendance problems most pertinent to a specific jurisdiction or culture. Such approaches could also help inform global policy review and dissemination and implementation practices for SA/A, discussed next.

## Global Policy Review and Dissemination and Implementation

One of the most significant challenges for researchers of SA/A has been effective dissemination and implementation of conceptualization, assessment, and intervention approaches into schools, physical and mental health agencies, and the corridors of policy makers. Reasons for this are myriad and may include lack of consensus among scholars, the complexity and heterogeneity of this population, disconnect between disciplines, school resistance, and substantial administrative, logistical, legal, and other restrictions uniquely faced by school officials (Kearney, 2003; Graeff-Martins et al., 2006; Keppens and Spruyt, 2017). With respect to the latter, for example, many schools have been restricted by zero tolerance laws that mandate specific sanctions for absenteeism that may displace clinical and other approaches (Gage et al., 2013). Exclusionary discipline policies, reporting guidelines, legal definitions of truancy, and disincentives for early school response likely play a role in this process as well (Marchbanks et al., 2015; Brouwer-Borghuis et al., 2019). Of course, many jurisdictions and countries have no legal or other policy regarding school absenteeism whatsoever (UNESCO, 2012). Furthermore, statewide truancy policies appear unrelated to chronic absenteeism levels and may actually be pernicious in that diverse students are subjected to more restrictive policies (Conry and Richards, 2018). Such policies also institutionalize the concept of truancy and thus color approaches taken for the problem (Spruyt et al., 2017).

Markussen and Sandberg (2011) noted that policy measures to address school absenteeism and dropout vary widely across countries, range from considerable to little impact, and are often affected more by economic shifts and labor markets. Still, the authors identified several policy measures across various countries that may have some impact on school absenteeism and dropout at system-wide levels, such as career guidance and counseling, income support for students, and vocational education and alternative educational programs. Markussen and Sandberg (2011) noted that these and other policy measures must be based on a deep understanding of local conditions, including the unique attributes of those with school absenteeism and dropout, as well as on a common commitment to develop better theory for addressing these issues within the context of each country. Global policy review with respect to school absenteeism must therefore focus on pruning counterproductive measures in addition to disseminating and implementing theoretical models that can be uniquely tailored to cross-cultural settings.

A multidimensional multi-tiered system of supports pyramid model of SA/A could be one such vehicle for policy review and dissemination. The model is consistent with whole-school reform models of education and eschews policies and practices that focus on exclusionary discipline (and unlawful school exclusion), immediate referrals to legal and other outside agencies, tacit acceptance of low-performing students who leave school, inflexible curricula, and rigid standardized testing (Kearney, 2016). In addition, the model and associated algorithms can be flexibly and practically tailored to idiosyncratic differences related to local norms, calendars, and educational practices. The model is designed to be inclusive, simple, and easily adaptable to extant modes of service delivery in schools, which are key parameters of successful dissemination and implementation (Lyon and Bruns, 2019). In addition, the multidimensional model may be well positioned because it can dovetail with (1) already existing school-based multi-tier frameworks devoted to academic performance, school climate/positive school culture, social and emotional competencies, and career readiness and (2) functional behavioral assessment practices, both of which are already understood and utilized by many school officials (Freeman and Simonsen, 2015; Eklund et al., 2019).

Lyon and colleagues (Cook et al., 2019; Lyon et al., 2019) iteratively adapted implementation strategies and recommendations from the healthcare sector to create a common nomenclature for such strategies that would be relevant to the educational sector. A total of 75 unique implementation strategies were compiled into several larger conceptual categories, which could apply generally to programs designed to promote school attendance and/or curb absenteeism (Lyon and Cotler, 2009). A full explication of these categories is beyond the scope of this article, but especially pertinent categories are briefly summarized next vis-à-vis a multidimensional model of SA/A.

One set of adaptations, “use evaluative and iterative strategies,” referred in part to understanding the unique aspects of a given school context to identify potential barriers to implementation (and which school officials can best facilitate implementation), execute changes incrementally, establish clear goals and outcomes, develop monitoring systems with fidelity, obtain student and family feedback, and adjust practices as needed. Perhaps the most common school-based barriers to MTSS-based models include lack of daily and consistent use as well as poor linkage of data with action (Leonard et al., 2019). A multidimensional multi-tiered system of supports pyramid model of SA/A can be, however, amenable to simple feedback mechanisms, reliance on data-based decision-making, incremental employment within each tier, multiple stakeholder involvement, and consultation practices that may erode such barriers (Forman and Crystal, 2015; Scott et al., 2019). In addition, many clinical procedures to address school absenteeism at Tier 2 can be adaptively administered by school-based social workers, psychologists, and counselors (Kearney, 2018, 2019).

Other sets of adaptations, “provide interactive assistance” and “adapt and tailor to context,” referred in part to using a centralized system within a district to assist in implementation, pair school personnel together, identify ways a new practice can best be adapted to a given school context, utilize experts to inform implementation efforts, and integrate educational and administrative data across schools. A key advantage of a multidimensional multi-tiered system of supports pyramid model of SA/A is that many schools already utilize MTSS or related tier-based principles as a centralized system and may thus be more equipped and willing to absorb school attendance/absenteeism into their frameworks. Use of student review boards, district-wide task forces, and similar existing mechanisms at the system level for truancy may be helpful in this regard as well (Bye et al., 2010). In addition, MTSS models of SA/A rely on attendance teams involving multiple school officials that can be informed by research-based findings (e.g., early warning systems and tier demarcations) described in this review (Kearney, 2016). Others have also appealed for better sharing of attendance and graduation rates across schools in a given district to identify which contexts have been more successful with respect to school completion and how certain practices can be extrapolated (DePaoli et al., 2015).

Other sets of adaptations, “develop stakeholder interrelationships,” “support clinicians,” and “engage consumers” referred in part to developing partnerships internal and external to a school (e.g., university and school board) for training purposes, adding different disciplines as needed, providing real-time data regarding student outcomes, constructing educational materials regarding new practices, engaging with families to become active participants, and utilizing media to reach large numbers of people. MTSS models commonly employ school-community/research partnerships involving varied professionals from mental health and youth-serving systems (Weist et al., 2018). In addition, Chu et al. (2019) recommended the use of researcher-designed, publically available platforms for deriving real-time attendance and related data that could be available to districts nationally and internationally. Many schools are also moving toward more standardized data collection systems with respect to basic performance outcomes (e.g., attendance, office disciplinary referrals, and course grades) in conjunction with new federal mandates (Egalite et al., 2017). As noted earlier, MTSS models also rely heavily on family and student engagement practices as well as educating parents about relevant school district policies regarding attendance and available resources (Kearney and Graczyk, 2014; Kearney, 2016).

Successful dissemination and implementation strategies for SA/A will likely have to include some level of absorption into what schools are already doing to address social, emotional, and behavioral competencies. Many/most schools already emphasize measurement, functional behavioral assessment, feasible multi-tiered approaches, and performance and student outcomes related to attendance, discipline, and academic progression (Lyon and Bruns, 2019). Schools are often motivated as well in an era of linked funding and mandates to improve attendance and graduation rates (DePaoli et al., 2018). In addition, school-based professionals often coordinate efforts with mental health, medical, legal, social service, and other outside agencies to help implement wide-ranging approaches for SA/A (Kearney, 2016). Successful dissemination and implementation strategies for SA/A will also have to involve adaptation to future changes in education and technology, a topic discussed next.

## Adaptability to the Future of Education and Technology

One of the biggest challenges for educators, researchers, clinicians, and others who study and address SA/A will be massive and rapid changes in education and technology over the next several decades. Any SA/A model will thus need to be pliable enough to be adapted not only to different cultures and countries but also to broad, systemic trends. This section discusses expected future trends in education and technology and then how a multidimensional, multi-tiered systems of support model for SA/A could be adapted. For brevity purposes, we group these trends into two broad categories: competency-based education and virtual learning (Kearney, 2016).

*Competency-based education* refers generally to mastery of academic and related material based on key benchmarks, and at a variable pace and timeline, rather than a strict focus on formal in-seat class time, examination scores, and credit accrual (Colby, 2017). Many schools in different countries have moved, or are moving toward, more holistic models of education that emphasize comprehension, innovation, conceptual connections, and critical thinking skills rather than simple recall and procedural steps (Jukes and Schaaf, 2019). In these authentic or ubiquitous learning environments, students are more apt to engage in project-, portfolio-, experiential-, and service-based activities to solve real-world problems, conduct experiments, interpret findings and literature, and make recommendations and presentations rather than simply taking multiple-choice tests, for example (Virtanen et al., 2018). Many such environments also emphasize personalized, customized learning and curricula, including core social and behavioral competencies, for preparing individualized adult and career readiness plans (Ekstrand, 2015; Taylor et al., 2017).

*Virtual learning* generally refers to online programming to deliver academic coursework and content (Brinson, 2015). Virtual learning environments are increasingly common at high school and postsecondary levels of education, but all future learning environments are expected to have at least some virtual component over the next several decades (Miron and Gulosino, 2016). Virtual learning environments can range in scope from adjunctive to hybrid to immersive in nature. An adjunctive scope may involve the introduction of greater technology into traditional classroom settings (e.g., game-based student-teacher interactions *via* tablets or smartphones; a hybrid or blended scope may combine online learning with direct (in-person) instructor contact; an immersive scope may involve a wholly digital network rather than a physical space that includes students from many different locations) (Hainey et al., 2016; Boelens et al., 2017; Xie et al., 2019). Virtual learning environments, particularly immersive ones, can also vary with respect to time of individual and group work and perhaps be modified more quickly *via* learning analytics than traditional classrooms (Williamson, 2017).

Future trends in education and technology have serious ramifications for contemporary SA/A models. Researchers’ traditional focus on outcomes such as percentage time missed from school as well as on concepts such as truancy or reluctance to attend school will need to be reconfigured in light of increasingly decentralized approaches to learning. In related fashion, researchers and others will likely need to reconsider traditional grade-level systems and academic calendars as schools increasingly modify the pace at which individual students learn, accrue credits (if relevant), and graduate.

A multidimensional multi-tiered system of supports model may be adaptable to these changes in education and technology. Indeed, various Tier 3 approaches for students largely disconnected or disengaged from school often focus on virtual, hybrid, project-based, and credit recovery and personalized learning approaches to provide alternative or blended pathways to adult and career readiness. In addition, many dimensional constructs associated with SA/A can dovetail with more dimensional aspects of the educational experience, including those linked to competencies, progression, completion, skill, and readiness for career paths. Finally, the model posed in this review is atheoretical, independent of academic timeline, and dexterous and malleable enough to accommodate rapid growth and immediate level change. Perhaps most importantly, the model emphasizes the promotion of school attendance and education in some form, an ever-present goal for all in this field.

## Conclusion

School attendance and school absenteeism remain important avenues of focus for many different professionals across education, mental health, public policy, and myriad other areas. As noted in Part 1 of this two-part review, though meant to be comprehensive, this article focused on the primary methods of differentiating school attendance problems. Many nuanced distinctions based on multilevel and other statistical modeling should be noted, and many special circumstances such as intense school violence, extreme poverty, and geopolitical factors likely override the distinctions mentioned here. However, the main goal was to provide a heuristic model to help spur the field toward reconciliation, common language, and advancement while considering important aspects of prevention and intervention, particularly within schools.

Also as noted in Part 1 of this two-part review, we offer deep appreciation to all those who have dedicated their time and careers to helping youth succeed in school and move to a more productive and healthy adulthood. The frameworks presented in this review are designed as looking glasses both into the past and future of SA/A and thus represent only a snapshot of the present state of affairs in this rapidly changing field. We look forward to learning about new and innovative developments in this field and hope that the ideas posed here offer some assistance.

## Author Contributions

All authors (CK, CG, PG, and MF) contributed to the writing and editing of the manuscript.

### Conflict of Interest

The authors declare that the research was conducted in the absence of any commercial or financial relationships that could be construed as a potential conflict of interest.
